# Experimental sleep deprivation as a tool to test memory deficits in rodents

**DOI:** 10.3389/fnsys.2013.00106

**Published:** 2013-12-13

**Authors:** Valeria Colavito, Paolo F. Fabene, Gigliola Grassi-Zucconi, Fabien Pifferi, Yves Lamberty, Marina Bentivoglio, Giuseppe Bertini

**Affiliations:** ^1^Department of Neurological and Movement Sciences, University of VeronaVerona, Italy; ^2^Mécanismes Adaptatifs et Evolution, UMR 7179 Centre National de la Recherche Scientifique, Muséum National d'Histoire NaturelleBrunoy, France; ^3^Neuroscience Therapeutic Area, UCB Pharma s.a.Braine l'Alleud, Belgium

**Keywords:** sleep, sleep-deprivation, memory, learning, cognitive impairment, Alzheimer's disease, rat, mouse

## Abstract

Paradigms of sleep deprivation (SD) and memory testing in rodents (laboratory rats and mice) are here reviewed. The vast majority of these studies have been aimed at understanding the contribution of sleep to cognition, and in particular to memory. Relatively little attention, instead, has been devoted to SD as a challenge to induce a transient memory impairment, and therefore as a tool to test cognitive enhancers in drug discovery. Studies that have accurately described methodological aspects of the SD protocol are first reviewed, followed by procedures to investigate SD-induced impairment of learning and memory consolidation in order to propose SD protocols that could be employed as cognitive challenge. Thus, a platform of knowledge is provided for laboratory protocols that could be used to assess the efficacy of drugs designed to improve memory performance in rodents, including rodent models of neurodegenerative diseases that cause cognitive deficits, and Alzheimer's disease in particular. Issues in the interpretation of such preclinical data and their predictive value for clinical translation are also discussed.

## Introduction

Everyone is aware of the detrimental effects of insomnia, and hundreds of scientific papers have documented the negative consequences of sleep loss. Experimental procedures of sleep deprivation (SD), both in humans and in animal models, have been widely employed to unveil various aspects of sleep function *per se* as well as to study the effects of sleep loss on subsequent brain function at the molecular, cellular, physiological, and cognitive levels.

The first reported experimental study on SD was conducted on puppies at the end of the 19th century (de Manaceine, 1894), followed by other pioneering reports on experimental animal insomnia, mainly in dogs (for a review see Bentivoglio and Grassi-Zucconi, 1997), and by the first formal human SD study (Patrick and Gilbert, 1896). In the following decades, the dog as animal model for SD was progressively replaced by the cat and later on by rodents, with the rat being the animal of choice up to this day. With the introduction of gene manipulation technology, wild-type and transgenic mice are increasingly used in SD studies.

Just like insomnia, experimental SD causes measurable deficits in a variety of cognitive tasks. These deficits can be used to elicit mild, transient cognitive impairment in an otherwise normal individual. Such challenge can be of translational relevance in preclinical and clinical studies aimed at evaluating the efficacy of symptomatic drugs designed to improve cognitive performance. This is of critical importance for Alzheimer's disease (AD), for which an evaluation of the predictive value of preclinical testing is urgently needed.

Several review papers have summarized evidence on SD-related cognitive impairments in humans (Walker, 2008; Killgore, 2010; Lim and Dinges, 2010), but much less attention has been devoted to comprehensive accounts describing comparable results in animal models. A recent review article describes the consequences of sleep loss on a variety of cognitive tasks in rodents (McCoy and Strecker, 2011). The present review has instead a specific focus on studies in which experimental SD in rodents is followed by assessments of memory functions; special attention is paid to the practical guidelines for the design of experiments in which SD is used to induce a transient memory deficit. Studies on the use of SD in the investigation of the effects of cognitive enhancers are also dealt with.

Rodents have been widely used in sleep research to study sleep architecture, as well as sleep homeostasis, circadian rhythms, and their neurochemical and molecular correlates. The idea to exploit the effects of SD on subsequent cognitive abilities is relatively recent, and the present in-depth analysis focuses on papers that include behavioral testing of animals following SD and on studies that explicitly and accurately describe the methodological aspects of the SD protocol and behavioral paradigm.

## Sleep, sleep states, and sleep deprivation in humans and in rodents

The organization of the sleep-wake cycle differs in humans and rodents. First, laboratory rats and mice are nocturnal animals, and therefore spend the majority of daylight time resting, while nighttime is the active phase of the day. Second, rats are typically polyphasic, i.e., show repeated episodes of sleep during a 12:12 h light/dark (L/D) cycle. However, they spend around 70–80% of the night in wakefulness, and 70–80% of daytime asleep (Timo-Iaria et al., 1970). The daily amount and circadian distribution of sleep in mice (which are more active than rats in standard laboratory conditions) are similar to those of rats, though they exhibit variability across strains (Mistlberger, 2005).

Sleep in mammals includes two states: rapid eye movement (REM) sleep and non-REM (NREM) sleep. These two types of sleep are defined by electrophysiological signs detected in humans by a combination of electroencephalography (EEG), electrooculography and electromyography. NREM sleep, which in humans is divided into four stages of increasing depth, is characterized by high-voltage, low-frequency (“synchronized”) wave activity and behavioral quiescence, with reduced activity in postural muscles. Besides the characteristic ocular movements, REM sleep (also known as paradoxical sleep) is defined by a low-voltage and high-frequency EEG pattern similar to that of wakefulness but, at variance with wakefulness, with suppressed vigilance and postural muscle tone. The loss of postural muscle tone during REM sleep is exploited in experimental SD procedures (see section Rem-Selective Sleep Deprivation).

NREM and REM sleep alternate in cycles, with NREM sleep preceding REM sleep epochs; NREM-REM sleep cycles have a fairly constant period, with a duration of 90 min in humans (Pace-Schott and Hobson, 2002). NREM-REM sleep cycles vary in duration as a function of the size of the animal and last about 12 min in the rat (McCarley, 2007).

All SD paradigms consist of partial or near-complete removal of sleep in an organism. Due to homeostatic regulation of sleep, SD causes a progressively accumulating sleep debt that results in increasingly greater efforts to maintain wakefulness over time.

With the exception of drug-induced insomnia, experimental SD in humans is invariably obtained by engaging subjects in a variety of activities that help maintain sufficient vigilance levels in order to avoid sleep. A crucial contribution in keeping subjects awake is, of course, motivation, whether provided by explicit rewards or by the knowledge of participating in a scientifically important activity. Furthermore, volunteer subjects know in advance about the duration of the experiment and are aware of the benign outcome of the experience.

These apparently self-evident considerations are very relevant when comparing experimental SD in humans and laboratory animals. By definition, human experimental SD is a “gentle,” non-threatening procedure, whereas SD protocols in animals are inevitably contaminated by a degree of stress that may even represent an important confound for the interpretation of results. Indeed, as it will be outlined below, much effort has been put into developing SD protocols with the least amount of stress and other aversive conditions.

Twenty-nine distinct methods to induce insomnia in rats and mice have been reviewed by Revel et al. (2009). Examples are stress-related models, such as environmental changes (e.g., cage exchange, introduction of aversive odors) or discomfort (e.g., exposure to cold temperatures or pain); pharmacological models (e.g., administration of caffeine, psychostimulants, modafinil, etc.); genetic models (e.g., DBA/2J mice, clock gene mutants, orexin/hypocretin overexpression, etc.). Most of these protocols (Revel et al., 2009) create an underlying condition that is incompatible with sleep (hence the term insomnia). The experimental SD techniques in the focus of the present review aim instead at preventing an otherwise sleepy animal from falling asleep.

An important distinction needs to be made between *total* and *partial* or *state-dependent* SD. In the former procedure, all sleep is prevented for the desired amount of time, independent of sleep state. In partial SD, on the other hand, a specific sleep state is selectively targeted for deprivation. In the vast majority of cases, partial SD is restricted to REM sleep, although selective NREM SD can also be accomplished. Sleep state selectivity, total duration and temporal pattern of SD are chosen on the basis of the particular function under study or the particular deficit to be elicited.

It is commonly accepted that, even when carefully implemented, SD protocols only approximate their nominal goals. For example, SD can never be truly “total” for extended periods of time, as episodes of “microsleep” (short episodes of intrusion of sleep into wakefulness lasting as little as a few seconds) become inevitable with the accumulation of sleep debt (Friedman et al., 1979; Durmer and Dinges, 2005). Also, selective deprivation of one sleep state inevitably affects the amount and distribution of the other sleep state.

Two additional manipulations of the temporal schedule of SD, namely sleep restriction and sleep fragmentation are worth mentioning. With sleep restriction, the amount of sleep can be reduced by a predefined amount thus mimicking the human condition of abnormally late “bedtime” and/or early “wakeup” hours over an extended period of time (Leemburg et al., 2010). In sleep fragmentation procedures, sleep can be prevented for repeated but brief epochs, both within a single resting period and across several days. For example, animals can be forced to walk on a treadmill for 30 s, and be allowed to rest for 90 s over an extended period of time [see for example (Tartar et al., 2006)]. The obtained repeated arousals, i.e., brief, transient increases in EEG frequency, resemble conditions frequently observed in humans, such as those associated with either extrinsic stimulation (e.g., noise) or intrinsic events (e.g., sleep apnea) (Bonnet, 2005).

## Sleep deprivation protocols in rodents

### Total sleep deprivation by gentle handling

The “gentle handling” (GH) procedure is based on a direct interaction with the experimenter, who actively keeps the animal awake, and is by far the most popular method of total SD in the rodent. The procedure aims at minimizing the spurious effects of stress and of forced locomotor activity imposed by other methods (see below). GH requires the constant physical presence of dedicated and fully trained experimenters with whom animals must be familiarized prior to the experiments. Animals, usually kept in their home cage during the SD procedure, are actively monitored by the experimenter, with or without the support of EEG and electromyographic recordings. The operator's task is to stimulate the animals just enough to keep them awake, whenever drowsiness or attempts to engage in a sleeping posture are observed, and/or if EEG signs of low-frequency activity appear. Two broad categories of stimulation can be distinguished: (1) passive exposure to external stimuli, (2) engagement in spontaneous exploratory and locomotor behavior.

1. In the case of passive exposure to external stimuli, the animal can be subjected to external stimulation, such as mild noises, tapping or gentle shaking of the cage, or by direct contact with the animal either through a soft brush or by hand. A certain degree of induced stress should always be taken into account as SD manipulations are often associated with elevated corticosterone levels (Longordo et al., 2009). Repeated handling *per se* causes alterations in locomotor activity and an increase in circulating corticosterone levels in comparison with undisturbed animals (Meerlo et al., 2008; Longordo et al., 2011). When comparing “mild” stimulation, such as tapping on the cage, to “stressful” direct handling of the animals at 30 min intervals, it was found that corticosterone levels in the former group were comparable to those of control animals, but were three times as high in handled animals (Kopp et al., 2006). In SD by GH studies, control experiments should be aimed at teasing out the effects of stress and of generic sensorimotor stimulation from the specific consequences of sleep loss. For example, SD by GH administered during the first 6 h of the resting circadian phase immediately after the acquisition of a fear conditioning paradigm affected the consolidation of fear memory, while an equivalent period of SD carried out during the active phase did not (Hagewoud et al., 2010). This result supports the hypothesis of a specific role of sleep loss on memory consolidation, rather than a non-specific effect of the handling procedures (but see Cai et al., 2009).
2. Protocols aimed at engaging exploratory activity may include the introduction of novel objects or nesting material in the cage (Tobler et al., 1997; van der Borght et al., 2006), which typically leads to active exploratory behavior and has been shown to help maintain wakefulness (Hairston et al., 2005). Stress caused by stimuli that elicit spontaneous exploratory behavior, reflected by corticosterone levels, is indistinguishable from that of controls, which would make the latter approach preferable, in principle (Kopp et al., 2006). On the other hand, stimulating the animal by enriching the environment in order to enforce wakefulness may in some cases represent a confound for subsequent behavioral and cognitive tests. For example, performance in the novel object recognition task (NOR, see below) may be altered by previous exposure to unfamiliar objects in the cage.

The choice of protocol and the intensity and frequency of stimulations are directly proportional to SD duration. In our experience, rats and mice are easily kept awake by mild stimulation for the first few hours, but direct handling becomes necessary beyond 5–6 h of SD. Adapting the amount of stimulation to each animal's instantaneous vigilance inevitably introduces variability among different animals but, on the other hand, presumably minimizes stress.

An experienced operator can administer GH to a small number of rodents at once (in our own lab, 6 would be considered the maximum), given that detection of sleep-seeking behavior and/or of sleep-related brain activity would be less effective as the number of monitored animals increases. When properly executed, SD by GH effectively suppresses nearly all sleep activity in rats and mice. In rats, SD by GH can reduce NREM sleep by 92% and REM sleep by 100% (Franken et al., 1991), even though the occurrence of microsleep needs to be taken into account. On the other hand, the method is labor-intensive and may be difficult to fully standardize across laboratories and even between operators within the same lab.

### Automated total sleep deprivation

Total SD can be administered by forcing specific patterns of locomotor activity, either continuously or as soon as the animal shows behavioral and/or electrophysiological signs of impending sleep. Several automated devices have been devised to make sleep impossible in rodents.

Continuously moving treadmills or rotating wheels are commonly used apparatuses to achieve total SD in animals (Borbely and Neuhaus, 1979). Another automatic method to enforce total SD is the “alternating platform.” The apparatus is made of two small platforms, placed in a water tank, which continuously and alternatively emerge from and are submerged by water, thus forcing the rat or mouse to a permanent motion in order to avoid contact with water. The method has been reported to completely abolish sleep (Pierard et al., 2007).

The obvious advantage of such procedures is that the quality and amount of stimulation can be standardized and made equal for all experimental animals. On the other hand, the concern that effects measured after the procedure may be a consequence of stress and fatigue rather than of sleep loss *per se* is higher in these paradigms than in GH.

In an attempt to control for the contribution of the SD-enforcing stimulation to results, Rechtschaffen and coworkers (Rechtschaffen et al., 1983; Bergmann et al., 1989; Rechtschaffen and Bergmann, 1995) introduced the “disk-over-water” (DOW) apparatus, which to this day is regarded as the reference automated technique for total SD. In this paradigm, one experimental and one control rat (or mouse) are housed on each side of a cage split in half by a divider, and with the floor replaced by a rotating disk suspended over a tank of shallow water. As soon as the experimental rat (or mouse) enters a “forbidden” vigilance stage, the disk starts rotating, forcing the animal to walk in the opposite direction to avoid falling into the water. The control animal is able to sleep whenever the experimental one is spontaneously awake and therefore the disk is not rotating. Thus, both the experimental and control animals receive the same mild sensory stimulation and a similar locomotor load. Inevitably, if both animals attempt to sleep at the same time, they are both woken up by the rotating disk. It is therefore unavoidable that controls are at least partially sleep deprived as well. In one typical total SD study (Everson et al., 1989), total sleep time was reduced by 91% in experimental animals, and by 28% in yoked controls, compared to baseline recordings.

It should be noted that the DOW method has typically been administered over extended periods of time (at least several days, and up to 1 month) to animals kept in constant light conditions. The L/L (light/light) condition rapidly disintegrates the diurnal sleep/wake cycle (Eastman and Rechtschaffen, 1983), thereby making sleep pressure homogeneous in both SD and control animals, independent of subjective circadian rhythmicity. However, while this manipulation allows a direct comparison between experimental groups, the disruption of the circadian rhythm adds complexity to the interpretation of any subsequent cognitive deficits. In the context of studies of cognitive functions, one could argue that for a relatively brief period of SD in the DOW apparatus the two animals should be kept at opposite light-dark phases. To our knowledge, there are no reports on the use of the DOW method for the relatively short SD periods (<24 h) typically adopted in cognition studies.

Another method of total SD in rodents is represented by the “grid over water” (Shinomiya et al., 2003). Placing a rat on a grid floor suspended over a tank filled with water significantly reduces total NREM and REM sleep time and increases sleep latency and wakefulness total time compared to rats placed on sawdust. This method has been proposed for the evaluation of the hypnotic properties of drugs (Shinomiya et al., 2003).

### REM-selective sleep deprivation

The striking differences in brain activity between sleep states have long represented a major focus for sleep research, and methods that allow selective deprivation of each sleep state (state-selective sleep deprivation, SS SD) have therefore received much attention.

In humans, SS SD performed with the aid of polygraphic monitoring allows selective deprivation of either REM sleep or deep NREM sleep (usually stages 3–4). Deprivation limited to superficial NREM sleep is impossible to accomplish in practice, as subjects would have to be woken up immediately upon falling asleep, which would be equivalent to total SD. While polygraphic monitoring has also been used in rodents, the sudden drop in muscle tone at the onset of REM sleep makes it possible to implement REM-selective SD in the rodent in the absence of electrophysiological recordings.

REM-selective SD can be easily obtained in rodents by means of the so-called “flowerpot method”. Initially employed to study sleep in cats (Jouvet et al., 1964), the procedure has since been adapted to deprive mice and rats of REM sleep. In the original version, the animal is placed in a water tank, on top of a small platform (traditionally an upside-down clay flower pot) slightly raised above the water surface (Morden et al., 1967; Mendelson et al., 1974). After some preliminary adaptation to the apparatus, the animal is left undisturbed in the water tank for the duration of the experiment. The platform allows the animal to crouch and to obtain NREM sleep *ad libitum*. However, at the onset of each REM period, the loss of muscle tone causes the neck to relax and the snout to touch the water, thus arousing the animal. As the duration of the SD period increases, the animal increasingly loses balance and falls in the water. The typical control is represented by an animal placed in a similar environment but on a platform sufficiently large to allow for a fully relaxed posture and hence REM sleep.

Because of its simplicity, the technique has been widely used, contributing to an understanding of the roles and mechanisms of REM sleep. It should be pointed out, however, that although the procedure primarily targets REM sleep, a substantial loss of NREM sleep has been also reported (Grahnstedt and Ursin, 1985; Machado et al., 2004). Furthermore, the procedure is accompanied by a non-negligible amount of stress (Revel et al., 2009) which may confound the interpretation of the results. In order to reduce stress, the protocol has been modified by placing multiple platforms in a larger tank, and by sleep-depriving several animals at once (van Hulzen and Coenen, 1981). Thus, animals are free to move around the cage, jumping from platform to platform, and to interact with their cohorts, thereby reducing the stress caused by immobility and social isolation (but see Suchecki et al., 1998; Machado et al., 2004).

As mentioned above, the flowerpot method allows to perform SS SD without the aid of electrophysiological recordings. Unfortunately, no comparable, straightforward technique for selective NREM SD is available. Where polygraphic recordings are available, SS SD in rodents can be implemented in a similar way as in humans. For example, upon entering a forbidden zone (either REM sleep or high-amplitude NREM), animals can be aroused in ways that are comparable to those used in GH, either by giving objects to play with or by introducing acoustic and tactile stimuli (Endo et al., 1997).

Another protocol introduced for REM SD is the “head-lifting method,” proposed to reduce some of the disadvantages encountered with the majority of REM SD methods (Datta et al., 2004). In this procedure, the beginning of each REM sleep episode is identified by observation of ongoing polygraphic records. From the adjacent room, the experimenter presses a mechanical lever within a few seconds from REM sleep onset, so that the animal's head is gently lifted and the animal is woken up. Using this method, an experienced operator can terminate REM sleep episodes within 3–5 s of their onset.

The head-lifting method is thought to minimize extraneous stress and physical activity and eliminate the need for the experimenter's physical proximity to the rat. During a 6 h recording session, the head-lifting method has been reported to successfully eliminate 90–95% of total REM sleep with no significant reduction of SWS (Datta et al., 2004).

## Sleep deprivation-induced memory impairment in rodents

As mentioned above, much effort has been devoted in humans to the study of the impact of SD on various cognitive domains, including vigilance and basic attentional processes, memory, as well as more complex real-world tasks.

While it is clear that all mammals share a fundamental physiological need for sleep and that prolonged SD has a dramatic impact on the organism, some important consequences of SD on high-level human cognition may well be beyond the reach of animal models. As with any other field of comparative study, reproducing in rodents the SD-induced deficits observed in humans is made difficult by the intrinsic differences between the species sleep-wake cycles. Thus, utmost care should be devoted to the choice of the behavioral/cognitive task and interpretation of the results to obtain meaningful insights from SD studies.

In rodents, investigations on the role of sleep loss and SD have focused on their effects on learning and memory performance (Tables 1, 2). Several examples are presented in the following paragraphs.

**Table 1:** **Synopsis of impairments in memory function after sleep deprivation in laboratory rats and mice**.

**Memory function**	**Test**	**TSD**			**REM-SD**			**SR**		**SF**
		**≥6 h**	**10–12 h**	**>1d**	**4–6 h**	**12–24 h**	**>72 h**	TSR^*^	REM-SR^*^	
Spatial memory acquisition	MWM			r, m	r	r	r	r, m		r
Spatial memory retention	RAM		r		r					
	MWM			r, m	r	r	r	r, m		r
Avoidance acquisition	RAM				r					
	Fear conditioning, avoidance test				r, m	r, m	r, m	r		
Contextual memory	Fear conditioning, avoidance test	m	m		r, m	r, m	r, m		r	
Working memory	Y-maze, RAM	r	r, m							m
Object recognition memory	NOR	r, m								m

* Brief and repeated sleep restriction episodes.

Abbreviations: m, r, tested in mice, rats, respectively; MWM, Morris water maze; NOR, novel object recognition; RAM, radial-arm maze; REM-SD, REM-selective sleep deprivation; REM-SR, REM-selective sleep restriction; SF, sleep fragmentation; SR, sleep restriction; TSD, total sleep deprivation; TSR, total sleep restriction.

**Table 2:** **Studies on sleep deprivation-induced memory impairment in laboratory rats and mice (1981–2012)**.

**PMID**	**References**	**Species**	**Strain**	**SD method**	**SD duration**	**SD time schedule**	**SD vs. training**	**Test**	**EEG monitoring**
22989412	Inostroza et al., 2013	r	LE	GH	80 min	Continuous	After	NOR	n
23238166	McCoy et al., 2013	r	LE	Activity wheel	18 h	Repeated for 5 days	After	MWM	n
23082139	Kumar and Jha, 2012	r	W	GH, rotating disk	6 h	Continuous	After	FC	y
22654204	Yang et al., 2012	r	SD	GH	4 h	Repeated for 7 days	After	MWM	y
22521334	Fernandes-Santos et al., 2012	m	Swiss	GH	6 h	Continuous	After	PM-DAT, FC, PA	n
22321457	Leenaars et al., 2012	r	W	Rotating wheels	3–12 h	Continuous	After	OT	n
22192378	Hajali et al., 2012	r	W	Flower pot	72 h	Continuous	Before	MWM	n
21677190	Walsh et al., 2011	r	SD	Flower pot	6 h	Continuous	After	MWM	y
21788501	Rolls et al., 2011	m	C57BL	Electric stimulation	30 s	Repeated for 4 h	After	NOR	y
21624432	Aleisa et al., 2011	r	W	Flower pot	24 h	Continuous	After	RAWM	n
21356250	Esumi et al., 2011	r	W	Flower pot	96 h	Continuous	Before	AT	n
21295147	Chowdhury et al., 2011	r	W	GH	6 h	Continuous	After	OT	n
21120129	Patti et al., 2010	m	EPM-M1	GH^a^; flower pot^b^	^a^6; ^b^72 h	Continuous	Before, after	PM-DAT	n
20717746	Xu et al., 2010	m	C57BL	GH	3 h	Repeated for 20 days	Before	MWM	n
20614860	Moreira et al., 2010	r	W	Flower pot	96 h	Continuous	Before	AT	n
20561181	Legault et al., 2010	r	SpD	Flower pot	4 h	Continuous	After	RAM	n
20495497	Zhao et al., 2010	r	SpD	Flower pot	72h	Continuous	Before	MWM	n
20394312	Alhaider et al., 2010	r	W	Flower pot	24 h	Continuous	Before	RAWM	n
20050994	Hagewoud et al., 2009	m	C57BL	GH	6–12 h	Continuous	Before	NART	n
19962429	Dubiela et al., 2009	r	W	Flower pot	96 h	Continuous	Before	PA	n
19850085	Ramanathan et al., 2010	r	SpD	GH	6 h	Continuous	Before	Ym	n
19847264	Vecsey et al., 2009	m	C57BL	GH	5 h	Continuous	After	FC	n
19794184	Cai et al., 2009	m	C57BL	GH	12 h	Continuous	After	FC	
19645967	Ward et al., 2009	r	FN	Treadmill	24 h	Continuous	Before	MWM	n
19643093	Ward et al., 2009	r	FN	Treadmill	24 h	Continuous	Before, after	MWM	n
19627456	Chang et al., 2009	r	W	DOW	5 d	Continuous	Before	MWM	n
19619610	Tian et al., 2009	r	SpD	Flower pot	6 h	Continuous	After	FC	n
19597374	Wang et al., 2009	r	SpD	Flower pot	72 h	Continuous	After	MWM	n
19542091	Li et al., 2009	r	SpD	Flower pot	48 h	Continuous	After	MWM	n
19444749	Palchykova et al., 2009	m	OF1	GH	6 h	Continuous	After	NOR	n
19193874	Bjorness et al., 2009	m	KOm	Treadmill	48 h	Continuous	During	RAM, PA	y
19186164	Halassa et al., 2009	m	KOm	GH	6 h	Continuous	After	NOR, FC	y
19014078	Novati et al., 2008	r	W	Rotating wheels	20 h	Repeated for 8 days	Before	FC	n
18985181	Kalonia et al., 2008	r	W	Grid over water	72 h	Continuous	Before	Active avoidance, MWM	y
18775445	Ruskin and Lahoste, 2008	m	C57BL	Flower pot	24 h	Continuous	Before	FC	n
18707010	Alvarenga et al., 2008	r	W	Flower pot	96 h	Continuous	Before, after	PM-DAT	n
18674519	Yang et al., 2008	r	SpD	Flower pot	120 h	Continuous	After	MWM	n
18329112	Perry et al., 2008	r	W	Flower pot	96 h^a^ and 18 h^b^	^a^Continuous and ^b^repeated for 21 days	Before	AT	n
18281713	Calzavara et al., 2009	r	SHR	Flower pot	96 h	Continuous	Before	FC	n
17920644	Silvestri and Root, 2008	r	SpD	Flower pot	6 h	Continuous	After	FC	n
17698177	Pierard et al., 2007	m	C57BL	Automated TSD	10 h	Continuous	Before	SA-WM	y
17157993	Fu et al., 2007	r	SpD	Flower pot	6 h	Continuous	After	FC	n
16876303	Silva et al., 2007	m	M	Flower pot	24 h	Continuous	Before	PA	n
16817877	Tartar et al., 2006	r	SpD	Treadmill	24–72 h	Continuous	Before	MWM	y
16423541	Palchykova et al., 2006	m	OF1	GH	6 h	Continuous	After	NOR	n
16376302	Chen et al., 2006	m	C57BL	Flower pot	24 h	Continuous	Before	FC	n
16325867	Ruskin et al., 2006	r	SpD	Flower pot	72 h	Continuous	After	MWM	n
16014798	Hairston et al., 2005	r	R	GH	6 h	Repeated for 5 days	After	MWM	y
15777764	Dubiela et al., 2005	r	W	Flower pot	96 h	Continuous	Before	AT	n
15763570	Silvestri, 2005	r	SpD	Flower pot	6 h	Continuous	After	FC	n
15582679	Legault et al., 2004	r	SpD	Flower pot	4/10 d	Repeated for 10 days	During	RAM	n
15582021	de Oliveira et al., 2004	r	W	Flower pot	72 h	Continuous	before	AT	n
15341794	Silva et al., 2004a,b	m	EPM-M1	Flower pot	72 h	Continuous	Before, after	AT	n
15317872	Su et al., 2004	r	SpD	Flower pot	12–24 h	Continuous	After	FC	n
15262203	Guan et al., 2004	r	SpD	GH	6 h	Continuous	Before	MWM	n
15182321	Ruskin et al., 2004	r	SpD	Flower pot	72 h	Continuous	Before	FC	n
15033349	Silva et al., 2004a,b	m	EPM-M1	Flower pot	72 h	Continuous	Before	AT	y
14960614	Datta et al., 2004	r	SpD	Head-lifting	6 h	Continuous	After	AT	y
14573548	McDermott et al., 2003	r	SpD	Flower pot	72 h	Continuous	Before	FC	n
12954399	Wang et al., 2003	r	W	Flower pot	24 h	Continuous	After	AT	n
12788510	Moreira et al., 2003	r	W	flower pot	96 h	Continuous	Before	AT	n
12773581	Graves et al., 2003	m	C57BL	GH	5 h	Continuous	After	FC	n
12644281	Smith and Kennedy, 2003	r	SpD	Flower pot	48 h	Continuous	After	AT	n
11809508	Dametto et al., 2002	r	W	Flower pot	96 h	Continuous	Before	AT	n
11527327	Le Marec et al., 2001	r	SpD	Flower pot	4 h	Continuous	Before	MWM	n
10958153	Bueno et al., 2000	r	W	Flower pot	96 h	Continuous	Before	AT	n
10866356	Kennedy et al., 2000	r	SpD	Flower pot	24–48–96 h	Continuous	After	AT	n
10857657	Beaulieu and Godbout, 2000	r	SpD	Flower pot	12 h	Continuous	Before	MWM	n
10837820	Prathiba et al., 2000	r	W	Flower pot	96 h	Continuous	Before	AT	n
10604833	Youngblood et al., 1999	r	W	Flower pot	96 h	Continuous	During	MWM	n
9619997	Smith et al., 1998	r	SpD	Flower pot	4–12 h	Continuous	After	RAM	n
9438789	Smith and Rose, 1997	r	SpD	Flower pot	4 h	Continuous	After	MWM	n
9035255	Youngblood et al., 1997	r	SpD	Flower pot	96 h	Continuous	During	MWM	n
8848497	Smith and Rose, 1996	r	SpD	Flower pot	4 h	Continuous	After	MWM	n
8788869	Gruart-Masso et al., 1995	r	W	Flower pot	5 h	Continuous	Before	AT	n
7800747	Bueno et al., 1994	r	W	Flower pot	24–72–96 h	Continuous	Before	AT e FC	n
1896504	Coll-Andreu et al., 1991	r	W	Flower pot	3–6 h	Repeated for 5 days	After	AT	n
3413257	Marti-Nicolovius et al., 1988	r	W	Flower pot	5 h	Repeated for 5 days	After	AT	n
3212058	Smith and Kelly, 1988	r	SpD	Flower pot	24–72 h	Continuous	After	AT	n
3174846	Ambrosini et al., 1988	r	W	Flower pot	3 h	Continuous	After	AT	
7163344	Harris et al., 1982	r	W	Flower pot	72 h	Continuous	Before	AT	n
6891076	van Hulzen and Coenen, 1982	r	W	Flower pot	72 h	Continuous	Before	AT	n
7178252	Smith and Butler, 1982	r	SpD	Flower pot	4 h	Continuous	After	AT	n
7053243	Kitahama et al., 1981	m	C57BR and C57BL	Flower pot	10 or 24 h	Continuous	After	AT	n

Abbreviations: AT, avoidance test; FC, fear conditioning; FN, Fisher Norway rats; GH, gentle handling; KOm, knock-out mice; LE, long evans rats; m, mice; MWM, Morris water maze; NOR, novel object recognition; OF1, Oncins France 1 mice; OT, operant task; PA, passive avoidance; r, rats; PM-DAT, plus-maze discriminative avoidance task; RAM, radial arm maze; RAWM, radial arm water maze; SD, sleep deprivation; SpD, Sprague Dawley rats; SHR, spontaneously hypertensive rats; W, Wistar rats.

### Spatial reference memory in the morris water maze

The study of spatial memory in rodents has focused on the innate ability to find and remember locations using any available, distant (allothetic) cues. This type of learning requires the formation of a spatial map of the environment. The most commonly adopted task to test spatial reference memory is the “Morris water maze” (MWM) (Morris et al., 1982; Morris, 1984). The basic setup consists of a circular tank filled with water rendered opaque by diluted inert paint. Animals are placed in the water and swim until they stumble upon and climb on a slightly submerged, and therefore invisible platform. On subsequent trials, animals learn to find the platform, always placed in the same position, more and more efficiently, aided by the visual cues available in the room. Training sessions consist of at least 4 trials (with each trial starting at a different location along the perimeter of the pool) and are typically repeated over a period of a few days. Performance can be assessed during training by measuring the distance covered and the time elapsed searching the escape platform. Importantly, in a final probe trial, the platform is removed from its usual location, and the time spent by the animal searching for the platform at the expected location is interpreted as a measure of *spatial, hippocampus-dependent memory* (Morris, 2001). In order to dissociate hippocampus-independent behaviors, the platform can be made visible in control trials, thereby allowing to control for sensory and motor abilities, visual acuity, motivation, and thigmotaxis (Packard and McGaugh, 1992).

Deficits in the ability to memorize the submerged platform's location have been reported as a consequence of both REM-selective SD and total SD by GH. The first study (Smith and Rose, 1996) was designed to establish the most appropriate time window following the daily training session for the administration of REM SD in rats. The most severe learning deficits were obtained when 4 h REM SD by the flowerpot method were administered beginning 5 h after the end of training. Interestingly, a recent report has documented a lack of substantial effects of 6 h REM SD preceding training in the MWM (Walsh et al., 2011).

A continuous 72 h REM SD with the flowerpot method administered to rats during their training period was shown to impair both the acquisition rate in the MWM and the ability to remember the position of the platform in the subsequent probe test (Zhao et al., 2010). Interestingly, the detrimental effects of pre-training REM SD on spatial learning and memory in the MWM are more severe in female than in male rats (Hajali et al., 2012).

In a study of SD-dependent alterations in molecular mechanisms of synaptic plasticity (Guan et al., 2004), rats were given 6 h of total SD by means of GH, starting at *lights-on* time (which effectively makes the length of the sleepless epoch longer when compounding the previous, normally active, wakefulness period). SD was immediately followed by a 12-trial training session in the MWM. The loss of sleep did not cause significant impairments in the animals' learning rate compared to controls. However, a probe trial without the platform performed 24 h later showed in the sleep-deprived animals a lack of preference for the expected platform location, suggesting an impairment of spatial reference memory despite improved performances over time (Guan et al., 2004).

Rats subjected to a 5-day period of total SD by the DOW method and trained in the MWM immediately afterwards were found to be impaired in spatial learning and even more severely impaired in a test of long-term spatial memory performed 12 h later (Chang et al., 2009). Brief epochs of total SD repeated over an extended period of time also cause deficits in the MWM. At the end of a 30-day period during which mice were sleep-deprived every day for 3 h by means of GH, the animals received 3 daily sessions of training and a test (probe) session 24 h afterwards, and showed significantly impaired spatial learning and spatial memory retention (Xu et al., 2010). Similarly, decrements in the acquisition of the platform location in the MWM have been reported as a consequence of 6 h SD which occurred repeatedly during a 4-day training period (Hairston et al., 2005).

The so-called “place learning set task,” a modified MWM task, is regarded as a rodent equivalent of human short-term memory tests such as the digit-span task (Whishaw, 1985). The procedure consists of pairs of identical trials, separated by a short delay (up to 1 min). Trial pairs are separated by a substantially longer delay (5–30 min) and the starting position of the platform changes from one trial pair to the next. Each session consists of a variable number of trial pairs (8–12) and the entire training consists of several daily sessions, with the platform location changing every day. This version of the MWM allows to probe distinct skills and memory types. Differences in escape latency between the two trials in each pair are taken as a measure of either short-term memory (Whishaw, 1985; Ruskin et al., 2006) or working memory (Youngblood et al., 1997, 1999; Yang et al., 2008), whereas performance delta between trial pair sets is ascribed to spatial reference memory. Overall performance gains across training sessions reflect the ongoing learning process of the general procedure, independent of the specific platform location (Youngblood et al., 1997).

A decrease in learning rate was observed in this modified version of the MWM after 72 h of REM SD administered before testing. The deficit has been interpreted as due to either poor spatial working memory or to slow learning of the task rules (Ruskin et al., 2006). With the same paradigm, severe impairment of spatial reference memory performance with no deficit of spatial working memory has been reported in animals repeatedly tested over a 4–5-day REM SD period (Youngblood et al., 1997, 1999; Yang et al., 2008).

Similarly, sleep fragmentation for 24 h obtained with the treadmill method caused deficits in the retention of spatial reference memory (tested 24 h after training) in spite of a normal learning curve, suggesting selective interference of prior sleep disturbances with the consolidation of spatial memories (Ward et al., 2009).

In yet another variant of the MWM, the so-called “delayed alternation navigation task,” animals are placed in the water always in the same spot, and the platform is hidden at one of two locations, alternating on each trial. A short REM SD (from 4 to 8 h) preceding the session was found to disrupt the animals' performance with no deficits in the conventional (allocentric) version of the task, suggesting that hippocampus-independent, frontal-like performance could be more susceptible to a short period of REM SD than hippocampus-dependent spatial memory (Beaulieu and Godbout, 2000; Le Marec et al., 2001).

Dissociations in the effects of total SD on spatial *vs.* non-spatial memory have recently been investigated with a novel task (Pierard et al., 2011). The apparatus consists of a square open field whose floor can be changed both in color and texture, and with a hole placed in each of its 4 corners. In the spatial-only version of the task, the animal learns to find food pellets hidden in one of two diagonally opposite holes, solely aided by visual cues external to the open field (allocentric). The floor remains unchanged and neutral with respect to the outcome of these trials. After a retention interval followed by an epoch of total SD, spatial memory is tested by placing animals in the same open field, with all 4 holes unbaited, and counting the number of visits to the previously rewarded holes. In the context-dependent version of the test, each of the 2 baited holes is associated with a floor of specific color and texture. During the test phase, only one floor is presented, and the number of visits to the associated hole is taken as a measure of context-dependent memory. Total SD for 10 h caused a deficit in the more complex contextual task, but not in spatial memory *per se* (Pierard et al., 2011).

### Spatial working memory and the radial arm maze

Both in humans and animals, working memory can be defined as the ability to store and manipulate the information necessary to accomplish cognitive tasks such as learning within one session, but not between different sessions (Baddeley, 1992; Dudchenko, 2004). This function can thus be distinguished from long-term memory because of its “transient” character. Spatial working memory, that is working memory for locations, has been assessed in rodents with a variety of tests. For example, the “spontaneous alternation task” and the “novel arm recognition task” exploit the animals' innate exploratory behavior, which leads to spontaneously alternate visits between arms in a maze on each trial, or to spend more time in the novel than in previously explored arms, without the use of behavioral reinforcers. Retaining the necessary information in order to successfully alternate between arms as well as spending more time in the novel arm compared to the previously explored arms is assumed to require intact working memory. While spontaneous alternation paradigms are considered more dependent on the frontal cortex (Verma and Moghaddam, 1996), spatial memory guided by external cues is highly dependent on the hippocampal formation (Winocur and Moscovitch, 1999; Yoon et al., 2008; Alhaider et al., 2010). Spatial working memory in rodents has been challenged with 6–10 h of total SD. In these studies, animals were habituated to the apparatus and then subjected to total SD before being tested in the maze. Total SD was shown to impair the animals' alternation rate as well as the time spent in the novel arm relatively to the previously explored arms (Pierard et al., 2007; Hagewoud et al., 2010).

A widely used procedure to assess both spatial working memory and spatial reference memory in rodents is the “radial arm maze” (RAM) (Olton and Samuelson, 1976). At odds with the previously described tests, in the RAM reward is adopted to motivate behavior. The maze consists of a central platform and eight arms originating from it. Several prominent extramaze visual cues are usually situated around the testing room. In the original procedure, animals are trained to find food pellets placed at the end of each arm. Since only one pellet per arm is available, the optimal strategy is to visit each of the eight arms only once, which requires to hold in memory which of the arms have already been visited (Olton and Samuelson, 1976).

Dissociations between spatial reference and spatial working memory can be investigated with the RAM using a paradigm in which four arms are baited while the other four never contain food (Legault et al., 2004). After a number of training sessions, visits to the unbaited arms are regarded as long-term reference memory errors, while repeated visits to previously baited arms are scored as working memory errors (see also Olton and Papas, 1979). REM-selective SD administered during the 4 h period immediately following a training session in the RAM was found to elicit a deficit in spatial reference memory but not in spatial working memory (Smith et al., 1998; Legault et al., 2004).

An alternative version of the RAM is the “win-shift paradigm,” which has also been used to assess hippocampus-dependent spatial memory. During daily training, animals are placed at the center of the maze and all arms are opened after a brief delay. When an animal enters a given arm, the doors to the other arms are closed. When the animal returns to the central platform, its door is also closed; after a delay, all eight doors are again opened and the animal can choose another arm. The routine is repeated until all baits have been obtained or until a maximum trial duration (10 min) is reached. The time taken by an animal to complete the RAM task each day and the number of baits eaten by an animal during a trial could be used as indicators of rate of learning. Learning rate in the win-shift paradigm is severely impaired when a period of 4 h REM SD occurred immediately after each daily session of training (Legault et al., 2010).

A hybrid of the RAM and the MWM, the “radial arm water maze” (RAWM) (Diamond et al., 1999; Alzoubi et al., 2009; Alhaider et al., 2010), has been adopted to explore spatial memory and the integrity of related hippocampal function after SD. A four- or six-arm maze is placed inside a circular water tank, with submerged walls almost reaching the surface, and an escape platform located at the end of one of the arms (goal arm). The procedure differs from a canonical MWM in that animals entering the wrong arm are forced to swim back to the central area and then swim into another arm until they eventually find the goal arm. In each trial of this task, the animal is placed in the water at the end of a non-goal arm and is allowed a predefined amount of time to reach the goal platform. Rats submitted to 12 h REM SD commit significantly more errors than controls in finding the hidden platform both during the acquisition phase of the task and during the short-term memory test administered 30 min after the end of the learning phase (Alhaider et al., 2010). Moreover, rats that successfully learn to find a submerged platform fail to locate it if they are tested at the end of a 24 h REM SD administered immediately after the training (Aleisa et al., 2011).

### Contextual or pavlovian fear conditioning or cued learning

The effects of SD on associative learning, i.e. the ability to form new or to modulate existing associations, have been extensively explored. One of the most commonly adopted paradigms is classical “fear conditioning” (FC). In the “cued” version of the paradigm, the animal is trained to learn the association between an initially neutral conditioned stimulus (CS, usually an auditory tone), and a biologically relevant unconditioned stimulus (US, usually a mild electric footshock). After a single pairing between the cue and the shock, the CS will predict the occurrence of the US and elicit a response similar to that caused by the US. In the “uncued” task, the association is established between the learning context and the US. Fear conditioning is considered a rodent model of declarative memory (Anagnostaras et al., 2001), and both contextual and cued learning are amygdala-dependent, whereas contextual learning is also hippocampus-dependent (Ehrlich et al., 2009; reviewed by Radulovic and Tronson, 2010). SD has been shown to interfere with the consolidation and acquisition of contextual FC but not of cued FC.

Selective REM SD preceding training has been repeatedly reported to impair contextual FC in rats (Ruskin et al., 2004; Tiba et al., 2008). In particular, control animals show a pronounced freezing response when placed in the same environment where they previously experienced electric shocks, whereas sleep-deprived animals fail to show such behavior. Similar negative effects on contextual fear memory have been reported after a chronic sleep restriction (rats kept awake for 20 h and allowed to sleep for 4 h over 3 consecutive days) preceding training (Ruskin and Lahoste, 2008).

Moreover, the critical time window for contextual FC has been identified as the 5 h immediately following 5 h of training. In fact, total SD by GH immediately after training has been shown to affect the consolidation of memory for contextual FC tested 24 h after training (Graves et al., 2003; Vecsey et al., 2009; Hagewoud et al., 2010). Interestingly, no impairment in memory for contextual fear has been found when the SD period was initiated 5 h after the end of training (Graves et al., 2003; but see Su et al., 2004).

An alternative FC protocol is the “inhibitory avoidance task,” which has been widely used to study learning and memory in rodents. This task employs a 2-way shuttle-box with a guillotine door placed between the 2 chambers. One chamber is illuminated, while the other is in the dark. In the training session, the animals are individually placed in the illuminated chamber, facing away from the guillotine door. When the animal spontaneously enters the darkened chamber, a foot shock is applied through the grid floor, and the animal is moved back into the lit, safe chamber. The procedure is repeated until the animal learns about the association and does not cross the opening for 2 min. In the test sessions, the animals are again placed in the illuminated chamber and free to walk into the dark chamber. The latency to enter the dark chambers is interpreted as a function of recall of the aversive association, so shorter latencies may indicate cognitive impairment. Differently from the FC paradigm where the CS and US are delivered independently from the animal's behavior, in the avoidance task shock delivery is contingent on the animal's response and is therefore considered to test operant or instrumental learning.

In the inhibitory avoidance paradigm, REM SD preceding training does not prevent the animal from forming the association, as shown by normal rates of acquisition of the correct response. On the other hand, compared to cage controls, SD animals showed shorter latencies in entering the dark chamber during the test trials performed 24 h later (Moreira et al., 2003; Silva et al., 2004a,b; Esumi et al., 2011). This behavior could depend on impaired recall of the association at the time of testing or, more likely, on a disruption of the long-term storage of the environmental contingency.

Conflicting results have been reported on FC learning when REM SD is administered after training. In one study, 6 h of REM SD caused performance impairments in rats tested immediately after the deprivation procedure (Datta et al., 2004; Silva et al., 2004b). In another study, performance was normal immediately after 72 h of REM SD, but was severely affected when re-tested one week later (Silva et al., 2004a).

Periods of 9–12 h and 17–20 h after training have been identified as critical time windows for avoidance learning (Smith and Butler, 1982; Smith and Lapp, 1986). Increases of REM sleep after learning, which are considered to reflect a homeostatic response to the increased demands for memory consolidation (see Walker and Stickgold, 2004 for a review), are observed during these temporal intervals and REM SD is considered to be maximally effective in impairing response acquisition if administered during such times. It is worth mentioning that also paradoxical facilitatory effects on retention performance have been reported in an avoidance learning task when 24 h REM SD was adminstered immediately after training (Gisquet-Verrier and Smith, 1989).

Finally, the “elevated plus-maze (EPM) discriminative active avoidance” (or plus-maze discriminative avoidance task, PM-DAT) paradigm has been used in mice as alternative to classical protocols of associative learning. This task has the advantage of evaluating at the same time two closely related behavioral phenomena, memory and anxiety, as well as locomotor activity (Silva et al., 1997; Silva and Frussa-Filho, 2000). The EPM consists of four arms standing at some distance from the floor, two of which offer no protection while the other two are surrounded by opaque walls, and are typically preferred by rodents. The time spent in the closed *vs*. open arms is considered a measure of high-anxiety trait. In this version of the EPM, every time the animal enters a previously selected enclosed arm, aversive stimuli such as light and noises are presented and persist until the animal leaves the arm. At a variable delay after the conditioning, during the test session, animals are placed in the same apparatus but receive no aversive stimulation. Time spent in the aversive or non-aversive closed arms, time spent in the open arms as well as the total number of entries in any arms are measured. Decreases in the amount of time spent in the aversive arm during 10 min-training, measured minute by minute, is interpreted as learning of the task, whereas memory is measured as the percent time spent in the aversive *vs.* non-aversive enclosed arms in the test session. A significant difference between the time spent in the aversive and non-aversive enclosed arms in the test session is considered to reflect information retention (Silva et al., 1997). This paradigm allows also an assessment of anxiety levels by the percent of time spent in the open arms.

REM SD has been reported to produce different effects on acquisition and retrieval of the task depending on its duration and on the species used. Seventy-two hours of REM SD preceding training have not been reported to affect the learning of the task in rats (Silva et al., 2004a; Alvarenga et al., 2008), whereas 96 h REM SD does impair learning in mice (Silva et al., 2004a; Alvarenga et al., 2008). On the other hand, in both studies a detrimental effect of REM SD have been documented on the consolidation and retrieval of the PM-DAT during the test session, both when it preceded and followed the training (Silva et al., 2004a; Alvarenga et al., 2008).

The experimental paradigms presented above are instances of delay conditioning: the US follows the CS at some specific delays or the CS continues to be present during the CS-US interval. Unlike delay conditioning, trace conditioning requires the CS to be a discrete event and to be separated by a temporal gap from the US (Pavlov, 1927). Hippocampus has a prominent role in learning tasks that require temporal processing of information (McEchron et al., 1998; Runyan and Dash, 2005). Importantly, trace conditioning, especially in the form of eyeblink reflex conditioning, has been widely investigated as a model of associative learning and declarative memory in rodents (Christian and Thompson, 2003). To our knowledge, only one study explored the effects of SD on trace conditioning (Chowdhury et al., 2011). The authors reported that 6 h of total SD by gentle-handling soon after training impaired rats' performance in a task of trace-conditioned memory, measured as the number of head entries into a fruit juice dispenser (US). The authors therefore concluded that SD given after training was able to impair the encoding of trace memory.

### Object recognition

The “novel object recognition” (NOR) test exploits the rodents' spontaneous preference for novelty to measure recognition memory. In the typical paradigm, animals are presented and allowed to familiarize with a set of behaviorally meaningless objects for a brief amount of time (e.g., 5–10 min). After a variable delay (from a few minutes to one or more days) the object set is presented again, but with one of the familiar objects replaced with a novel one. The time spontaneously spent by the animal exploring the novel object, relative to the familiar one(s) is taken as a measure of recognition of the previously seen objects. It has been proposed that memory for objects in rodents is similar to episodic memory in humans (Dere et al., 2004) and relies upon peri- and post-rhinal regions rather than the hippocampus (Winters et al., 2004), if the spatial location of the objects is not changed.

Total SD by GH for 6 h following the acquisition phase in the NOR test severely impairs object and location recognition in a complex scene in a later test (Palchykova et al., 2006; Halassa et al., 2009). Interestingly, when SD was administered 6 h after the acquisition phase (thus allowing the animals to sleep undisturbed afterwards), no discrimination deficits were observed (Palchykova et al., 2006). Moreover, when GH occurred during the dark (activity) phase, no recognition memory deficits were observed in the subsequent test session (Palchykova et al., 2009).

Assessment of episodic memory in rodents relies on paradigms in which animals are required to bind the memory of an object to a spatio-temporal context (Kart-Teke et al., 2006). In such tasks, after few training trials, animals learn to remember not only the identity and location of the previously encountered object, but also the temporal order of object presentation. It has been reported that a brief (80 min) total SD by GH, following the acquisition phase in a modified NOR test, impairs rats' ability to consolidate and retrieve the memory for space, identity, and temporal order of presentation of an object (Inostroza et al., 2013).

### The effects of sleep recovery

A period of recovery sleep after SD is obviously considered beneficial to cognitive function. Relevant questions, however, remain open on the nature of such benefits.

In an instrumental learning study (Dubiela et al., 2005), one group of rats was deprived of REM sleep for 96 h, trained in an inhibitory avoidance apparatus immediately afterwards, and tested 30 min after the end of training. Performance was compared to that of rats allowed to recover for 24 h after REM SD and before training. The rate of acquisition was not affected by SD or by sleep rebound, but performance during the test differed across groups. In particular, SD animals entered the aversive chamber after a significantly shorter latency, compared to non-deprived rats, and animals allowed a period of sleep recovery showed an intermediate memory performance (Dubiela et al., 2005, 2010). The findings suggest that the avoidance deficits observed as a consequence of SD suffered immediately before training were due to inefficient/incomplete encoding of the memory association between the environment and the aversive experience.

Sleep recovery also improves recall of information acquired prior to the SD epoch. In a recent study (Wang et al., 2009) rats were subjected to 72 h REM-selective SD at the end of a 5-day training period in the MWM. The ability to recall the trained platform location was found to be impaired when tested immediately after SD, and the deficit was regarded as disruption of hippocampus-dependent spatial memory. When animals, however, were allowed to sleep for 18 h before the test, their performance turned out similar to that of control animals. This indicates that REM SD had no effect on the long-term memory trace consolidated over the training days. Rather, the results should be ascribed to deficits in *memory recall*, arguably due to the general disturbance of SD on cognitive function.

In both scenarios, SD appears to interfere with the animal's cognitive set rather than on the memory trace. In the former case, impaired cognition prevents encoding, while in the latter it prevents retrieval.

## Can drugs reverse SD-induced memory impairments?

**Figure 1:**
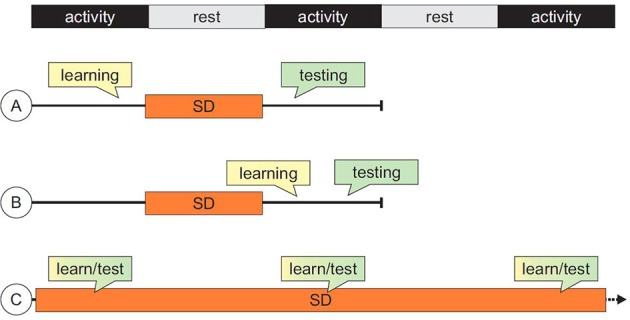
**Summary of typical sequences of events in studies of cognitive impairments induced by sleep deprivation (SD)**. (A) SD follows the acquisition or training phase and precede testing session(s); (B) both training and testing sessions are carried out *after* the SD epoch; (C) for tasks in which performance can be measured during training itself, sequences of training/SD can be administered repeatedly, allowing the assessment of long-term learning curves.

As described in the previous paragraphs, a transient memory deficit can be quite easily obtained in both rats and mice by means of SD. One of the crucial distinctions between the available paradigms is represented by the temporal relationship between the SD procedure and the animal's learning/testing experience (Figure 1). Depending on the adopted protocol, the observed memory deficits could be explained as the result of either impaired acquisition/consolidation or information retrieval. Consequently, drug-related effects would have to be attributed to mechanisms involving different aspects of the mnemonic experience.

The possibility to reverse SD-induced cognitive impairments through the administration of a variety of compounds (e.g., cognitive enhancers such as modafinil and donepezil, stimulants such as caffeine and nicotine, melatonin, vitamin E, etc.) has received some attention in recent years. In human healthy volunteers, positive effects of modafinil and caffeine in maintaining attentional and executive control performance during prolonged SD have been demonstrated (Wesensten et al., 2005; Killgore et al., 2009). On the other hand, chronic administration of donepezil (5 mg) has been shown to improve subjects' performance both in a visual short term memory and in a verbal episodic memory task after 24 h of total SD (Chuah et al., 2009; but see Dodds et al., 2011).

The effects of the non-amphetamine, wake-promoting agent modafinil in restoring memory performance after SD have been extensively studied both in mice and rats. The decrement in performance in the RAWM after 3 days of REM SD in rats was prevented by administration of modafinil for 1–3 days (He et al., 2011). Beneficial effects of modafinil administered prior to training were reported also in maintaining the inhibitory avoidance response after 96 h of REM SD in rats (Moreira et al., 2010). Modafinil, administered after training and SD, restored spatial working memory performance in a spontaneous alternation task (Pierard et al., 2007) and compensated contextual memory deficits in mice after 10 h of total SD (Pierard et al., 2011). Acute nicotine treatment also prevented retention deficits in a RAWM after 24 h of REM SD in rats (Aleisa et al., 2011). Significant pro-cognitive effects of melatonin (Chang et al., 2009) and vitamin E (Alzoubi et al., 2012) on memory impairments induced by SD in rats have also been reported.

Experiments testing the efficacy of drugs in reversing the transient memory deficits caused by SD could thus provide reliable models in drug-discovery preclinical studies for the treatment of dementia-related cognitive decline. To date, treatments of AD and other dementias rely on the administration of anticholinesterase therapy (e.g., donepezil, rivastigmine, galantamine) or NMDA receptor antagonists (i.e., memantine) whose effects at a symptomatic (cognitive and behavioral) level and as neuroprotective agents are still under debate (Bullock, 2002; Francis et al., 2005). The availability of rodent models of transient memory impairments would allow to specifically test the efficacy of drugs in ameliorating memory deficits and to study the duration of such improvements. In this context, the choice of the appropriate protocol of challenge, drug administration, and memory test deserves special attention not only in view of a translation to human patients, but also in relation to the specific aspect of memory impaired by SD (for example, learning impairment *vs.* deficit in the retrieval of previously acquired information).

## Concluding remarks

The available evidence here reviewed indicates that appropriately conducted SD protocols reliably cause memory deficits in rodents. Behavioral control conditions included in many studies offer convincing dissociations in support of the notion that SD-induced cognitive impairments are not simply the result of generic fatigue, stress, or lack of motivation. Rather, the results of properly controlled experiments suggest that deficits are caused by the disruption of specific high-level cognitive functions.

Moreover, certain functions are more prone to being disrupted by SD than others, and consistent experimental results may be difficult to obtain for some cognitive domains. For example, as previously reported, SD exerts a marked negative effect on hippocampus-dependent spatial memory consolidation tested in the MWM as well as on contextual fear memory tested in the shuttle-box, whereas no effect of sleep loss has been reported on cued fear memory. Interestingly, lack of significant deficits and even paradoxical performance improvements of SD have been also documented (Marti-Nicolovius et al., 1988; Smith, 1996; Tian et al., 2009).

Importantly, we find that the majority of studies employ SD as a tool to investigate the neurobiological mechanisms subserving sleep and sleep loss. Thus, the assessment of SD-induced cognitive deficits, rather than being the focus of the study, is often treated as an internal control of the success of the SD procedure, in view of subsequent molecular and neurophysiological evaluations. As a consequence, less attention is devoted to the subtleties of the experimental manipulations and consequent cognitive alterations. Thus, protocols that interfere with information encoding are used almost interchangeably with protocols that challenge information retrieval. The importance of dissociating the various cognitive processes underlying task performance is central when testing the efficacy of pro-cognitive drugs. For example, a different impairment is caused by protocols in which SD is administered *after training* but *before testing* and protocols in which SD *precedes* both learning and performance measurements, or in which epochs of SD are intermixed with training/test sessions (Figure 1). And yet, the 3 paradigms have been used to obtain “memory deficits” without any further connotation of the concept. Overall, the available data indicate that, while inducing memory deficits by SD in laboratory rodents is relatively straightforward, conceptualization of the effects of the different available protocols requires further testing and critical interpretation.

An example of a high-priority experimental use of SD is the preclinical testing of cognitive enhancers for mild cognitive impairment (MCI) and AD. MCI is considered to be a transitional phase between normal ageing and clinically probable, very early AD (Petersen, 2004). Currently available transgenic models of AD dementia have so far failed to fully replicate the phenotype of the human disease. For example, not all amyloid precursor protein transgenic mice become cognitively impaired, despite the presence of abundant plaques (Westerman et al., 2002). On the other hand, there are reports of cognitive impairment naturally occurring in aged laboratory rodents (Barnes and McNaughton, 1985; Gallagher et al., 1993), especially in the memory domain, but with striking interindividual variability in performance (Gallagher et al., 1993, 2003). It may be interesting, in principle, to apply SD protocols to transgenic animals and to measure the severity of subsequent memory impairments in order to quantify the additional costs, if any, of pathological changes and behavior challenge. SD as cognitive challenge offers several benefits over other experimental procedures, in that its effects are transient, it is relatively easy to administer in a standardized fashion, it avoids pharmacological manipulations of cognition (drug-induced deficits). SD as cognitive challenge may therefore provide a promising preclinical model of MCI and a useful tool to study cognition enhancing drugs. Despite its inherent limitations and the specific concerns raised above, SD may prove especially successful in the context of translational research by allowing direct comparisons between preclinical studies and investigations in humans: whatever role sleep and sleep loss may ultimately play in cognition, such role is conserved between rodents and humans.

### Conflict of interest statement

The authors declare that the research was conducted in the absence of any commercial or financial relationships that could be construed as a potential conflict of interest.

## Abbreviations

AD: Alzheimer's disease
CS: conditioned stimulus
DOW: disk over water
EEG: electroencephalography
EPM: elevated plus maze
FC: fear conditioning
GH: gentle-handling
L/L: light-light
MCI: mild cognitive impairment
MWM: Morris water maze
NOR: novel object recognition
NREM: non-rapid-eye movement sleep
PM-DAT: plus maze discriminative avoidance task
RAM: radial-arm maze
RAWM: radial-arm water maze
REM: rapid-eye movement
SD: sleep-deprivation
SS SD: state-selective sleep-deprivation
US: unconditioned stimulus.
